# TRANSPARENCY AND REPRODUCIBILITY IN THE NEUROIMAGING OF AGING

**DOI:** 10.1093/geroni/igz038.088

**Published:** 2019-11-08

**Authors:** Kendra L Seaman

**Affiliations:** Duke University, Durham, North Carolina, United States

## Abstract

In concert with broader efforts to increase the reliability of social science research, there are several efforts to increase transparency and reproducibility in neuroimaging. The large-scale nature of neuroimaging data and constantly evolving analysis tools can make transparency challenging. I will describe emerging tools used to document, organize, and share behavioral and neuroimaging data. These tools include: (1) the preregistration of neuroimaging data sets which increases openness and protects researchers from suspicions of p-hacking, (2) the conversion of neuroimaging data into a standardized format (Brain Imaging Data Structure: BIDS) that enables standardized scripts to process and share neuroimaging data, and (3) the sharing of final neuroimaging results on Neurovault which allows the community to do rapid meta-analysis. Using these tools improves workflows within labs, improves the overall quality of our science and provides a potential model for other disciplines using large-scale data.

